# Reliable Identification of Vehicle-Boarding Actions Based on Fuzzy Inference System

**DOI:** 10.3390/s17020333

**Published:** 2017-02-09

**Authors:** DaeHan Ahn, Homin Park, Seokhyun Hwang, Taejoon Park

**Affiliations:** 1 Department of Information and Communication Engineering, Daegu Gyeongbuk Institute of Science and Technology (DGIST), 333 Techno jungang-daero, Hyeonpung-myeon, Dalseong-gun, Daegu 42988, Korea; daehan@dgist.ac.kr; 2 Research Institute of Engineering & Technology, Hanyang University, 55 Hanyangdaehak-ro, Ansan, Gyeonggi-do 15588, Korea; hominpark@gmail.com; 3 Department of Interdisciplinary Engineering System, Hanyang University, 55 Hanyangdaehak-ro, Ansan, Gyeonggi-do 15588, Korea; hsh2438@naver.com; 4 Department of Robotics Engineering, Hanyang University, 55 Hanyangdaehak-ro, Ansan, Gyeonggi-do 15588, Korea

**Keywords:** driver identification, fuzzy inference system, vehicle-boarding actions, inertial sensors, driving while distracted

## Abstract

Existing smartphone-based solutions to prevent distracted driving suffer from inadequate system designs that only recognize simple and clean vehicle-boarding actions, thereby failing to meet the required level of accuracy in real-life environments. In this paper, exploiting unique sensory features consistently monitored from a broad range of complicated vehicle-boarding actions, we propose a reliable and accurate system based on fuzzy inference to classify the sides of vehicle entrance by leveraging built-in smartphone sensors only. The results of our comprehensive evaluation on three vehicle types with four participants demonstrate that the proposed system achieves 91.1%∼94.0% accuracy, outperforming other methods by 26.9%∼38.4% and maintains at least 87.8% accuracy regardless of smartphone positions and vehicle types.

## 1. Introduction

Manipulating a smartphone while driving causes distraction behind the wheels, and hence, is a serious threat to the safety of the driver and others [1,2,3,4]. Studies conducted by the United States Department of Transportation [5] indicate that nearly 20% of reported vehicle incidents, leading up to 9 fatalities and 2000 crashes everyday, involve distracted drivers caused by use of their smartphones while driving. Considering the serious risks of driving while distracted (DWD) on public safety, many countries enforced laws to ban smartphone usage for all drivers [6]. However, there still exist a number of drivers who resist to stop using their smatphones, thus causing DWD, since it is almost impossible for police officers to monitor each and every drivers on the road [7].

To avoid such a risk, it is crucial to activate DWD prevention measures if a user is driving a vehicle. According to the literature [8,9,10,11], drivers can be identified by using built-in smartphone sensors to tell both vehicle-boarding directions (left or right) and seated rows (front or rear) based on various driving-related actions, e.g., swinging legs and rotating a body when entering a vehicle, as well as events taken when, e.g., sitting in a vehicle, wearing a seat-belt, pressing a pedal, and/or running over a pothole.

However, these solutions are insufficient because sensory features associated with driving-related actions and events can only be accurately extracted if the user explicitly follows the predefined norms and regulations on smartphone positions (or poses) and vehicle-boarding behavior. For example, the approaches based on body rotation features [9,11] require a user to enter the vehicle holding the smartphone inside a trouser pocket without behaving unexpectedly (e.g., abruptly swinging their legs, holding the smartphone in non-trouser pocket such as bag and hand). Violating such restrictions causes the features to be distorted or overridden by noisy information, which significantly degrades the system performance. To overcome these shortcomings, it is important to develop a *reliable* solution that guarantees a high level of accuracy as well as deals with all variants of vehicle-boarding anomalies.

Motivated by this need, we propose a system, called VEhicle-boarding-action Recognition and Identification exploiting FuzzY inference (VERIFY), which reliably and accurately recognizes the vehicle-boarding directions solely using inertial measurement unit (IMU) sensors of smartphones. In particular, VERIFY: (1) extracts unique sensory features monitored from a broad range of complicated vehicle-boarding actions; and (2) processes them with a fuzzy inference system (FIS) [12,13] tailored to DWD prevention to maximize the reliability. Consequently, VERIFY effectively relaxes the pose restrictions of existing solutions, thereby allowing users to freely carry their smartphones, i.e., in pockets, bags or hands, with no limitations in vehicle types.

From our extensive experiments, we observe that entering from the left side of the vehicle generates a clockwise moving trajectory while the opposite yields a counter-clockwise trajectory. Moreover, such trajectory patterns are consistently monitored despite the presence of computational errors caused by unstable sensing precisions and clock drifts [14]. VERIFY constructively exploits this feature to compute the moving trajectory of a smartphone when entering the vehicle, and extract invariant directional information from this trajectory.

To exploit this unique feature, VERIFY first triggers its subsequent processes when the user stands beside a vehicle and keeps on collecting sensor readings until the user’s hip touches a seat. Second, it computes a moving trajectory using the collected data. Third, it locates three representative points along the trajectory using line approximation techniques. These points represent instances of *standing* (when the user is standing beside the vehicle), *turning* (when the user’s body is rotated to enter the vehicle), and *hip-strike* (when the user is seated). Lastly, it determines the vehicle-boarding direction from these three points by processing them with our proposed FIS. The use of FIS enables VERIFY to accurately detect the turning and and hip-strike points, which in turn maximizes the reliability of differentiating the left- and right-side boarding in the presence of complicated vehicle entrance activities commonly found in real-life environments.

Our comprehensive experiments on three different vehicle types with four participants demonstrate that VERIFY achieves the accuracy of 91.1%∼94.0% in identifying a broad range of complex boarding actions, and hence, outperforms other methods by 26.9%∼38.4%. VERIFY also maintains high accuracy (at least 87.8%) for all three smartphone positions (pockets/bags/hands) and three vehicle types (subcompact/mid-sized/full-sized) while others suffer from significant degradation of accuracy if smartphones are not in the preferred positions.

The main contributions of this paper are as follows: we (1) identify unique sensory features consistently monitored from a broad range of complicated vehicle-boarding actions; (2) present VERIFY that reliably and accurately classifies the sides of vehicle entrance by employing FIS to constructively utilize these unique features; and (3) evaluate a prototype of VERIFY by comparing its performance with other methods via real-world experiments.

The remainder of this paper is organized as follows. Section 2 details the architecture of our proposed system while Section 3 descrives the design of FIS for the purpose of DWD prevention. Section 4 and Section 5 present the experimental setup and the results of performance evaluation, respectively. Finally, the paper concludes with Section 6.

## 2. System Architecture

VERIFY utilizes built-in IMU sensors of smartphones. The smartphone we use is equipped with an InvenSense’s MPU-6500 6-axis device as the IMU sensor. This device has programmable full-scale ranges of up to ±2000${}^{\circ }/s$ (3-axis gyroscope) and ±16 g (3-axis accelerometer) as well as on-chip 16-bit ADCs, programmable digital filters, and a precision clock with 1% drift.

In designing VERIFY, we make the following two assumptions. First, users may freely carry their smartphones (in pockets, bags or hands) when entering the vehicle. Second, remote vehicle starters and door openers are not available. The second assumption can be easily met since remote vehicle door openers are rarely used [15].

**Figure 1:**
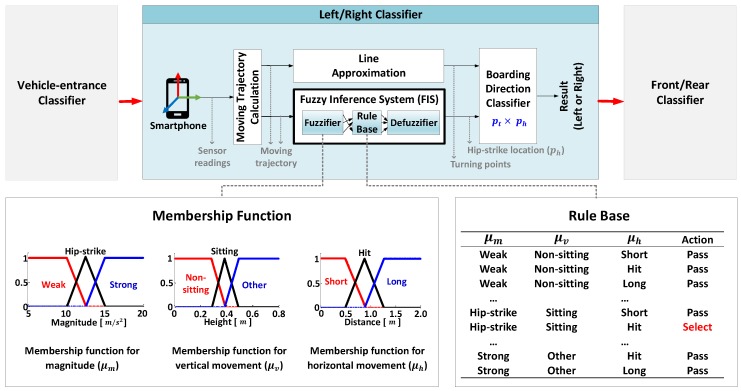
The proposed system consisting of three building blocks for: (1) detecting a vehicle-entrance event; (2) determining the side (left or right) of vehicle-boarding; and (3) differentiating the seated row (front or rear), where the left/right classification employs a fuzzy inference system (FIS) to maximize the reliability.

Based on these assumptions, VERIFY works as shown in Figure 1. Since vehicle-boarding actions are always preceded by approaching and standing by the vehicle, it first triggers a *vehicle-entrance classifier* and detects when a person is about to enter the vehicle by applying, e.g., the methods of [11,16]. In [11], it hinges on the fact that electromagnetic field (EMF) fluctuations get stronger when entering the vehicle compared to other similar daily activities, due mainly to special materials of vehicles and engines causing EMF to vary dramatically; these EMF spikes can only be detected when the smartphone is close to the vehicle since every commercial vehicle must pass a test to limit the EMF emission [17]. In contrast, [16] solely uses IMU sensors to detect subsequent changes from walking to standing and sitting states.

When such vehicle entering events are detected, VERIFY initiates the process of determining the vehicle-boarding directions (left or right) by activating a *left/right classifier*. After that, it subsequently executes a *front/rear classifier* to differentiate the seated row (front or rear) by exploiting, e.g., the method of [16]; it classifies the seated row based on the fact that starting the vehicle causes stronger in-vehicle EMF fluctuations to occur around the front seat where electronic components are densely populated. More specifically, it computes a rate of EMF changes for each time window and generates a bin-based frequency histogram, which exhibits a long-tailed (bell-shaped) distribution at the front (rear) row.

**Figure 2:**
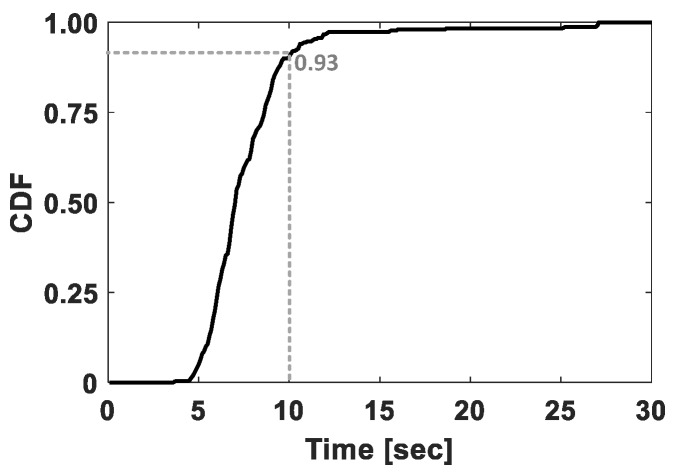
Cumulative Distribution Function (CDF) of time elapsed from the point of standing beside a vehicle to the point of being seated in the vehicle.

That said, VERIFY identifies the vehicle-boarding directions as follows. First, it collects sensor readings from IMU sensors embedded in a smartphone for a predefined period of time. Figure 2 plots a Cumulative Distribution Function (CDF) of time elapsed from the point of standing beside the vehicle to the point of being seated. Based on this, we set the duration of data collection to 10 seconds to ensure at least 93% of all sitting activities to be completely captured. Using these collected data, VERIFY calculates the moving trajectory of the smartphone by applying the method of Altitude Heading Reference System (AHRS) [14,18]. AHRS first converts the local sensor coordinate system, which continuously varies over time depending on smartphone orientations, into the reference coordinate system, in which *x* and *y* axes are in parallel with the ground plane of the global (Earth’s) coordinate system. This means that the *z* axis is perpendicular to the ground plane. As a result, the reference coordinate system effectively separates the moving trajectory into vertical and horizontal components.

**Figure 3:**
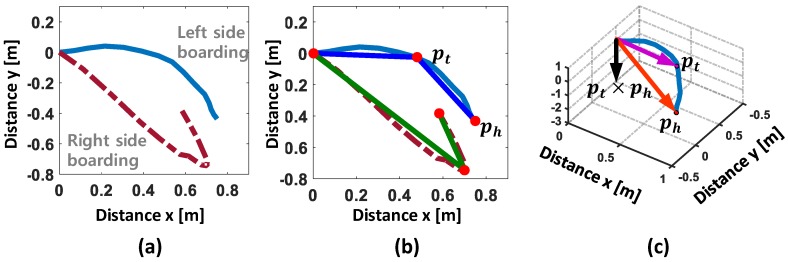
Process of boarding direction classification: (**a**) moving trajectories; (**b**) computing representative points by applying line approximation; (**c**) determining the boarding direction by computing $p_{t}\times p_{h}$.

Typical moving trajectories corresponding to two kinds of boarding directions are illustrated in Figure 3a. We note that the directions of moving trajectories found when the user enters the vehicle from the left side show clockwise curves while those from the right side portraits counter-clockwise curves. From this, the vehicle-boarding directions can be differentiated with three representative points along the trajectory, i.e., standing, turning, and hip-strike points as described in Section 1. To detect these points, one may simply execute line approximation by using the method of cubic Bézier curve least square fitting [19] that approximates the curved line to one or more straight lines. Figure 3b shows the results of applying this method, in which two red circles are located at the start and end of the moving trajectory, indicating standing and hip-strike points, respectively, while the third (middle) circle is located at or adjacent to the point where the turning movement occurs.

As shown in Figure 3c, VERIFY uses these three points to compute $p_{t}=\left[p_{t,x},p_{t,y},0\right]$ and $p_{h}=\left[p_{h,x},p_{h,y},0\right]$ on the horizontal plane of the reference coordinate system, each representing a vector from the standing to the turning point and a vector from the standing to the hip-strike point, respectively. The classification process then computes a cross product, $p_{t}\times p_{h}$, and determines the vehicle-boarding direction by looking at the sign of cross product, i.e., negative and positive signs indicating the left and right sides, respectively.

## 3. Design of Fuzzy Inference System

**Figure 4:**
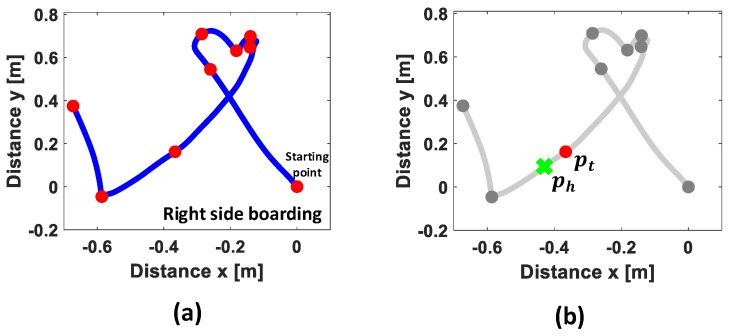
Inference of $p_{t}$ and $p_{h}$ from the complex moving trajectory by applying: (**a**) line approximation; (**b**) FIS.

If the moving trajectory has a simple shape as depicted in Figure 3a, VERIFY can easily and accurately determine the vehicle-boarding direction by plugging in the line approximation method in Section 2. However, in reality, it is not uncommon for VERIFY to encounter complex moving trajectories due to arbitrary and unexpected user behavior. For example, a user may take off a bag containing a smartphone from the shoulder before entering the vehicle or move the smartphone to a cup-holder after being seated. These activities that take place right before or after entering the vehicle contributes to forming a very complicated trajectory. This in turn produces multiple turning points, if using the line approximation, as illustrated in Figure 4a, making it very difficult to distinguish the vehicle-boarding directions.

To tackle this problem, we propose to design FIS that aims to reliably locate the hip-strike point, $p_{h}$ along the trajectory where the user’s hip touches the seat, by exploiting unique sensory features. Once $p_{h}$ is identified, VERIFY selects, as $p_{t}$, the turning point that took place right before $p_{h}$, as illustrated in Figure 4b.

After making hundreds of observations, we found that: (1) an actual hip-strike produces a spike on accelerometer readings; and (2) the hip-strike can only be monitored after certain lengths of vertical and horizontal movements from the standing point. Thus, FIS takes as inputs magnitudes of accelerometer readings, denoted as $\mu_{m}$, and vertical and horizontal moving distances, denoted as $\mu_{v}$ and $\mu_{h}$, respectively, at each point of the moving trajectory. It then analyzes the input data to determine $p_{h}$.

As shown in Figure 1, the proposed FIS (inside the left/rigit classifier) is comprised of a fuzzifier, a rule base, and a defuzzifier. The fuzzifier feeds the input to corresponding fuzzy sets consisting of three membership functions for $\mu_{m}$, $\mu_{v}$, and $\mu_{h}$, respectively, all of which are constructed empirically. Specifically, based on the experimental data collected from real vehicles, we design three membership functions and set parameters for them as described below. First, the membership function for $\mu_{h}$ is represented by using three linguistic terms, {*Weak*, *Hip-strike*, *Strong*}. From our experimental data, the average magnitude of accelerometer readings when the hip-strike takes place is 12.6 m/s${}^{2}$, so we use this value to set a boundary between *Weak* and *Strong* as well as specify a range of *Hip-strike* to two times the standard deviation of accelerometer readings, i.e., ±2.4 m/s${}^{2}$, to cover 95% of readings in case of a normal distribution. Also, among various shapes for the membership function, we choose a triangular shape with 50% overlapping according to [20]. Second, the membership function for $\mu_{v}$ represents the height difference monitored while entering the vehicle and uses three linguistic terms, {*Non-sitting*, *Sitting*, *Other*} where *Sitting* with a shape of 0.391 ± 0.094 m (average ± range) is defined as a boundary between *Non-sitting* and *Other*. Finally, we represent the horizontal displacement using three linguistic terms, {*Short*, *Hit*, *Long*} to define the membership function for $\mu_{h}$. Based on experimental results, we set a threshold between *Short* and *Long* to 0.89 m and fuzzify the displacement using 0.89 ± 0.38 m as the boundary value.

Once the inputs are fuzzified into values between 0 and 1, the rule base shown in Figure 1 generates the results based on 27 rules (three linguistic terms for each of three membership functions), each producing either of {*Pass*, *Select*}. In particular, it outputs *Select* when the fuzzifier provides *Hip-strike*, *Sitting*, and *Hit* for the magnitude, height, and horizontal displacement values, respectively. Otherwise, it chooses *Pass*.

Next, FIS implements a defuzzifier that combines the results from the rule base and generates a result of whether to select the point or not. Among well-known defuzzification methods FIS adopts the weighted average method [21], which effectively reduces the computational overhead. Figure 4b illustrates the result after applying FIS. It infers $p_{h}$ (green cross) on the moving trajectory and selects the previous turning point of $p_{h}$ as $p_{t}$. Finally, VERIFY (i.e., the boarding direction classifier in Figure 1) classifies the vehicle-boarding direction by computing the cross product, $p_{t}\times p_{h}$.

## 4. Experimental Setup

We compared the performance of VERIFY with three other smartphone-alone methods to determine the boarding direction: Motion Analyzer (MA) [16] to analyze the user’s motion based on the moving trajectory; Entry Swing (ES) [9] to compute the entry-leg-swing found when a user enters the vehicle; and Side Detection (SD) [11] to use the body rotations captured with gyroscopes. We implemented prototypes of these methods on Samsung Galaxy S5 smartphones [22] running Android${}^{\mathrm{TM}}$ and supporting 50Hz sampling rate for IMU sensing.

We recruited 8 participants as shown in Table 1 and conducted experiments in three vehicles types: subcompact car (Accent, Hyundai Motors Co., Seocho-gu, Seoul, Korea), mid-sized car (Sonata, Hyundai Motors Co., Seocho-gu, Seoul, Korea), and full-sized car (Grandeur, Hyundai Motors Co., Seocho-gu, Seoul, Korea) [23]. Moreover, we evaluated the system performance under two kinds of user behavior: (1) three normal cases (associated with the pocket, bag, and hand position, respectively), in which a user enters with neither smartphone manipulation nor position change, and (2) an extreme case, in which a user randomly locates the smartphone between three positions (pocket, bag and hand) as well as performs unusual actions such as dropping or throwing the smartphone. We collected a total of 720 data sets that consist of 360 data sets for each of left- and right-side entrance.

## 5. Performance Evaluation

**Figure 5:**
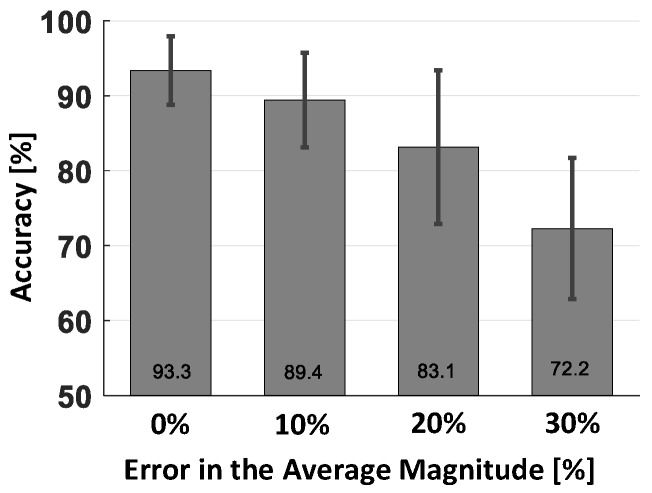
The performance of VERIFY by varying parameter values, i.e., by adding 0%, 10%, 20% and 30% errors to the membership functions of FIS.

We first evaluated the performance of VERIFY by varying its FIS parameters. That is, after determining average magnitudes of three membership functions from our data sets, we built four configurations of membership functions, each with different FIS parameters generated by adding 0%, 10%, 20% and 30% errors to the average values. Figure 5 shows that the best configuration (0% error) achieves the highest accuracy of 93.3% and that the accuracy keeps getting degraded as the degree of mismatch becomes higher (i.e., 89.4%, 83.1% and 72.2% accuracies for 10%, 20% and 30% errors, respectively). This reveals that it is important to properly set FIS parameter values although small errors (5%∼10%) in these values would be acceptable.

**Figure 6:**
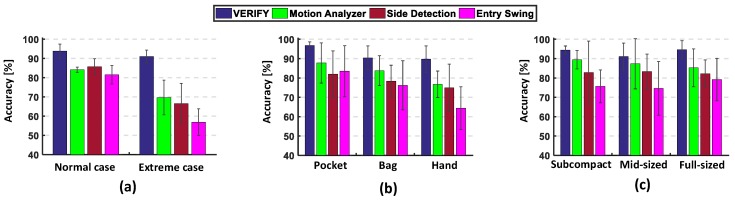
Experimental results on accuracy: (**a**) for normal and extreme cases; (**b**) by varying the position of smartphone on the body; (**c**) for three vehicle types.

Next, we compared the performance of VERIFY and others for each of the two cases as shown in Figure 6a. In the normal case, the average accuracy of VERIFY, MA, SD and ES are 93.8%, 84.1%, 86.6% and 81.6%, respectively, which means VERIFY has 7.2%∼12.2% higher accuracy than others. As expected, the performance gap gets much larger in the extreme case. That is, VERIFY, MA, SD and ES achieve the accuracy of 91.1%, 69.7%, 66.4% and 56.8%, respectively. This clearly indicates that VERIFY outperforms MA, SD and ES by 21.4%, 24.7% and 34.3%, thanks to its capability to intelligently recognize and remove irrelevant and/or noisy part of the moving trajectory.

We also evaluated the accuracies of three methods by varying: (1) the position of smartphone on the body and (2) the vehicle type as shown in Figure 6b,c, respectively. The results in Figure 6b reveal that all of MA, SD and ES suffer from significantly degraded accuracy in certain smartphone positions; that is, while they have over 80% accuracy when smartphones are held in pockets, their average accuracy drops to 79.9%, 76.6% and 70.2% in other positions, which are unacceptable for use in our daily lives. This undesirable performance degradation is due mainly to their incomplete system designs only considering the pocket position. In contrast, VERIFY maintains high accuracy for all smartphone positions, i.e., the average accuracies of 96.8%, 90.4% and 89.7% for the pocket, bag, and hand positions, respectively. Moreover, as shown in Figure 6c, VERIFY achieves 94.6%, 91.0% and 94.4% accuracies for subcompact, mid-sized, and full-sized vehicles, respectively, while MA, SD and ES have the average accuracies of 87.3%, 82.7% and 76.5%, respectively. These results clearly demonstrate the reliability/robustness of VERIFY against smartphone position variations regardless of the vehicle types.

**Figure 7:**
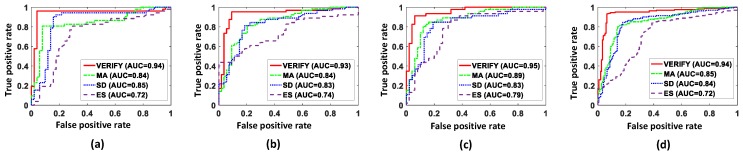
Receiver Operating Characteristic (ROC) curves and Area Under the Curve (AUC) values for: (**a**) subcompact vehicles; (**b**) mid-sized vehicles; (**c**) full-sized vehicles; and (**d**) all vehicles.

Finally, we evaluated VERIFY and others using Receiver Operating Characteristic (ROC) and Area Under the Curve (AUC) metrics [24]. An ROC curve is widely used to visualize the performance of a binary classifier where the curve is generated with true positive rates against false positive rates under various threshold settings. By using 360 data sets for left-side entrance as a true condition and the rest of 360 data sets for right-side entrance as a false condition, the true positive is when the system predicts positive (or left-side) results for the true condition while the false positive is when the system predicts positive results for the false condition. With the ROC curve, one can evaluate the performance of a classifier by computing an area under the ROC curve, or AUC, where AUC close to 1 indicates that the classifier is able to differentiate the target phenomena perfectly from the others while AUC ≤ 0.5 indicates that the classifier is meaningless. Figure 7 presents the results of ROC and AUC evaluation for each type of vehicles. VERIFY achieves AUC of 0.94, 0.93, and 0.95 for subcompact, mid-sized, and full-sized vehicles, respectively, which collectively yields an aggregate AUC of 0.94. By contrast, aggregate AUC values of MA, SD and ES are 0.85, 0.84 and 0.72, respectively. This reveals that VERIFY outperforms MA, SD and ES in terms of classification accuracy.

According to [9,11,16], MA, SD and ES achieved the accuracies of 98%, 90% and 91% for vehicle entrance classification, respectively, and 99%, 100% and 95% for front/rear classification, respectively. Nevertheless, the accuracy of an entire system integrating all three components drops to about 80% because a driver can be accurately identified only if it simultaneously succeeds in determining vehicle entrance, left or right, and front or rear, and hence, the left/right classification serves as the critical path significantly degrading the system accuracy. By contrast, VERIFY allows the system to achieve over 90% accuracy in identifying the driver thanks to its high accuracy of left/right classification.

## 6. Conclusions

In this paper, we presented a fuzzy inference-based system, called VERIFY, that accurately determines the vehicle-boarding direction (left or right) with restrictions on neither smartphone positions nor users’ boarding behavior. By developing a fuzzy inference system tailored to our needs, we were able to reliably identify whether or not a user is entering from the driver’s side. The results of comprehensive evaluation demonstrated that VERIFY outperforms other methods by 26.9%∼38.4% in terms of accuracy and reliably maintains high accuracy regardless of smartphone positions and vehicle types.

As a future work, we will equip VERIFY with adaptive methods such as [25] to provide reliable operations for each individual because it is important to consider various human behavioral factors (e.g., subject weight, height, age, and so on) and individual habits. Moreover, although we initially developed VERIFY for the purpose of identifying vehicle-boarding directions, the methodology proposed herein can be generalized to the inference of arbitrary behavior.

## Figures and Tables

**Table 1:** Participant information.

Subject No.	Gender	Age	Height (cm)	Weight (kg)
S1	M	29	173	65
S2	M	26	173	77
S3	M	29	178	85
S4	M	22	171	60
S5	F	22	158	48
S6	F	22	172	70
S7	F	23	163	48
S8	F	23	160	54

## Abbreviations

The following abbreviations are used in this manuscript:

AHRS	Altitude Heading Reference System
AUC	Area Under the Curve
CDF	Cumulative Distribution Function
DWD	Driving While Distracted
EMF	Electro Magnetic Field
ES	Entry Swing
FIS	Fuzzy Inference System
IMU	Inertial Measurement Unit
MA	Motion Analyzer
ROC	Receiver Operating Characteristic
SD	Side Detection
VERIFY	VEhicle-boarding-action Recognition and Identification exploiting FuzzY inference
